# Soil microbial community variation among different land use types in the agro-pastoral ecotone of northern China is likely to be caused by anthropogenic activities

**DOI:** 10.3389/fmicb.2024.1390286

**Published:** 2024-05-22

**Authors:** Zhaokai Sun, Chongzhi Sun, Tongrui Zhang, Jia Liu, Xinning Wang, Jing Feng, Shucheng Li, Shiming Tang, Ke Jin

**Affiliations:** ^1^Key Laboratory for Model Innovation in Forage Production Efficiency, Ministry of Agriculture and Rural Affairs, Institute of Grassland Research, Chinese Academy of Agricultural Sciences, Hohhot, China; ^2^School of Grass Academy, Qingdao Agriculture University, Qingdao, China; ^3^Anhui Science and Technology University, College of Agriculture, Huainan, China; ^4^Department of International Cooperation, Chinese Academy of Agricultural Sciences, Beijing, China

**Keywords:** land-use type, soil microbes, soil environment factors, microbial networks, local scale, bacteria and fungi

## Abstract

There are various types of land use in the agricultural and pastoral areas of northern China, including natural grassland and artificial grassland, scrub land, forest land and farmland, may change the soil microbial community However, the soil microbial communities in these different land use types remain poorly understood. In this study, we compared soil microbial communities in these five land use types within the agro-pastoral ecotone of northern China. Our results showed that land use has had a considerable impact on soil bacterial and fungal community structures. Bacterial diversity was highest in shrubland and lowest in natural grassland; fungal diversity was highest in woodland. Microbial network structural complexity also differed significantly among land use types. The lower complexity of artificial grassland and farmland may be a result of the high intensity of anthropogenic activities in these two land-use types, while the higher structural complexity of the shrubland and woodland networks characterised by low-intensity management may be a result of low anthropogenic disturbance. Correlation analysis of soil properties (e.g., soil physicochemical properties, soil nutrients, and microbiomass carbon and nitrogen levels) and soil microbial communities demonstrated that although microbial taxa were correlated to some extent with soil environmental factors, these factors did not sufficiently explain the microbial community differences among land use types. Understanding variability among soil microbial communities within agro-pastoral areas of northern China is critical for determining the most effective land management strategies and conserving microbial diversity at the regional level.

## 1 Introduction

Agro-pastoral ecotones consist of farmlands and grasslands that are important for agricultural production and animal husbandry in China. They also form ecological barriers and play critical roles in climate regulation and soil and water conservation (Zhu et al., 2007; Liu et al., 2009; Seddon et al., 2016). In the late 20th century, China enacted reforestation policies that led to widespread tree planting in these regions, which has resulted in a greater variety of land use types nationwide (Bryan et al., 2018; Liu X. et al., 2023). Although these land use changes have had important impacts on the structure and function of ecosystems (Wang et al., 2004; Verchot, 2010), the mechanisms of their ecological impacts remain unclear.

Soil microorganisms, consisting mainly of bacteria and fungi, play important roles in belowground biodiversity and in subsurface ecosystem functions, including organic matter decomposition, carbon (C) storage, nutrient cycling and redistribution, soil respiration, soil aggregate formation, and plant growth regulation (van der Heijen, 2008; Fowler et al., 2013; Schuur et al., 2015; Wu et al., 2023). Many studies have explored the effects of land use changes on soil microorganism communities in agro-pastoral areas (Kocyigit and Demirci, 2012; Lange et al., 2015; Yang et al., 2015); however, most have focused on grasslands and farmlands, whereas few have investigated artificial grasslands and woodlands, particularly in terms of belowground microbial communities (Peters et al., 2019; Felipe-Lucia et al., 2020).

The composition and diversity of soil microorganism communities are influenced by many factors (Philippot et al., 2023), including the types of vegetation within different land use types. Due to differences in vegetation types, certain plant inter-root microorganisms can enhance plant nutrient acquisition (Huang et al., 2014), stress tolerance (de Vries et al., 2020; Song et al., 2021; Schmitz et al., 2022), and help plants adapt to changing ecological conditions (Trivedi et al., 2020), which ultimately leads to differences in soil microbial diversity and composition. The complex ecological networks formed by microorganisms can also be influenced by changes in land use type. For example, a comparison of microbial network structures in tropical rainforest and rubber forest soils showed that the microbial structure of rubber forest soils had greater complexity and stability (Lan et al., 2022). A high degree of agricultural intensification can simplify soil bacterial and fungal network structures (Banerjee et al., 2019). Drought conditions have been shown to have a more pronounced negative effect on the stability of bacterial network structures than on that of fungal network structures (de Vries et al., 2018). Studies have reported significant differences in plant species, tillage intensity, and soil moisture under different land use types within the agro-pastoral ecotone of northern China (Bai et al., 2022; Liu et al., 2022). Together, these findings suggest that microbial community composition, diversity, and network structure vary considerably among different land use types in the agro-pastoral ecotone.

Changes in microbial communities among different land use types within agricultural and pastoral areas of northern China may be associated with various soil properties (Furtak and Galazka, 2019; Huang et al., 2023) or anthropogenic activities (Banerjee et al., 2019). Soil provides a substrate for microorganisms, and changes in its basic physicochemical properties such as pH and electrical conductivity, as well as soil moisture and levels of nutrients such as C, nitrogen (N), phosphorus (P), and potassium (K) can influence the structure of soil microbial communities (Furtak and Galazka, 2019). These properties can be influenced by human activities such as ploughing, irrigation, and fertilizer application, and may be altered to a greater extent following changes in land use type (Six et al., 2004; Geisseler and Scow, 2014). For example, soil erosion due to ploughing can affect bacterial extracellular polysaccharides and fungal hyphae, thereby reducing microbial abundance and diversity (Sae-Tun et al., 2022), and organic and chemical fertilizer inputs can alter soil aggregate structure and other soil physicochemical properties that affect soil microbes (Geisseler and Scow, 2014).

The objective of this study was to investigate the soil microbial communities within different land use types in the agro-pastoral ecotone of northern China, which may contribute to recommendations for rational land use planning in this region. To eliminate the interference of geographical, climatic, and soil factors, we selected a study site in the city of Hohhot, Inner Mongolia, China (Supplementary Figure S1 and Supplementary Table S1). We hypothesized that the composition, diversity, and network structure of soil microorganisms would differ considerably among different land use patterns, and that soil microbial community structural differences would partly result from human activities, but mainly from differences in soil properties in different land use types.

## 2 Materials and methods

### 2.1 Study site

This study was conducted at the Agro-pastoral Ecotone Experimental Station, Grassland Research Institute of the Chinese Academy of Agricultural Sciences (40°35′N, 111°46′E) in Hohhot, Inner Mongolia, China. The study site has a mesothermal continental monsoon climate, with a mean annual temperature of 6.7°C and mean annual precipitation of 400 mm. Rainfall occurs mainly in July and August. The soil type is predominantly tidal soil.

### 2.2 Soil sampling and testing

Land use was classified into five types: shrublands dominated by *Caragana korshinskii Kom*, woodlands dominated by *PopulusL*, artificially managed grasslands dominated by *Leymus chinensis*, natural grasslands dominated by *Stipa capillata*, and agricultural fields consisting of maize (*Zea mays*) cropland. Of these, only artificial grasslands and farmlands involved water and fertilizer addition and tillage management (see Supplementary Table S1 for more details). On July 25, 2023, we established ten sample plots (1 m × 1 m) as biological replicates of each land use type, with a spacing of ≥10 m. The litter and humus layers were removed, and the upper soil layer (0–20 cm) was sampled using a soil auger at three randomly selected locations per plot; these samples were mixed to obtain a single composite sample. Thus, a total of ten composite samples were obtained per land use type. We removed visible stones, animal and plant residues, roots, and other substances from each sample, and then sieved the sample through a 2-mm mesh. The sieved soil samples were sealed in sterile plastic bags, placed in an ice box, and transported to the laboratory. Each soil sample was divided into two parts, for soil physicochemical property analyses and DNA extraction, respectively.

### 2.3 Soil properties analyses

Soil moisture content was measured gravimetrically by drying at 105°C until a constant weight was achieved. Soil pH and electrical conductivity were measured in a soil–water slurry (1:2.5, w/v) as described previously (Widdig et al., 2020). Soil organic C (SOC) and total N (TN) were quantified using the dichromate oxidation and Kjeldahl digestion methods, respectively (Qiu et al., 2018). Alkaline N (AN) decomposition was measured colorimetrically using ultraviolet spectrophotometry and the indophenol blue method (Hu et al., 2021). Soil available P (AP) and available K (AK) were extracted with 0.5 M sodium bicarbonate and 1 M ammonium acetate, and measured by molybdenum blue spectrophotometry and flame photometry, respectively (Cui et al., 2023). Soil microbial biomass C (MBC) and microbial biomass N (MBN) were determined using the chloroform fumigation–extraction method.

### 2.4 DNA extraction and high-throughput sequencing

Soil genomic DNA was extracted using the FastDNA Spin Kit for Soil (MP Biomedicals, Irvine, CA, United States), following the manufacturer’s instructions. All DNA in 0.5 g of soil was extracted according to the recommended amount for the instrument. DNA quality was evaluated via 1% (w/v) agarose gel electrophoresis. Soil bacterial and fungal community compositions were determined by high-throughput sequencing on a cloud-based platform at Majorbio (Shanghai, China). The V3–V4 hypervariable regions of bacterial 16S rRNA genes were amplified using the 338F (5′- ACT CCT ACG GGA GGC AGC AG-3′) and 806R (5′-GGA CTA CHV GGG TWT CTA AT-3′) primer set (Xu et al., 2016). In fungi, the internal transcribed spacer 2 (ITS2) region was amplified using the ITS1F (5′-CTT GGT CAT TTA GAG GAA GTA A-3′) and ITS2R (5′-GCT GCG TTC TTC ATC GAT GC-3′) primer set (Karlsson et al., 2014). Polymerase chain reaction (PCR) amplification was performed using a GeneAmp PCR system (Model 9,700; Thermo Fisher Scientific, Waltham, MA, USA).

The raw data sequences were processed and analyzed using QIIME2 (Bolyen et al., 2019) based on the workflow provided at https://qiime2.org. Briefly, to obtain the amplicon sequence variant (ASV) table, quality control of the raw sequencing data was performed using the DADA2 (Callahan et al., 2016) plug-in and clustered based on 100% shared identity. The taxonomy of bacterial and fungal phylotypes was identified using the silva138/16s_bacteria (Quast et al., 2013) and unite8.0/its_fungi (Nilsson et al., 2019) databases, respectively. Finally, we obtained 3,318,073 bacterial sequences and 4,373,406 fungal sequences, which were classified into 40,525 and 7,910 distinct ASVs in bacteria and fungi, respectively.

### 2.5 Analysis of microbial community structure and correlation with soil properties

We analyzed α-diversity parameters such as the Chao1 and Shannon indices using the mothur ver. 1.30 software. Differences in mean α-diversity values between two independent groups were analyzed using the Wilcoxon rank-sum test with the *stats* package in the R ver. 4.3.2 software (R Core Team, Vienna, Austria). The Bray–Curtis dissimilarity index was used to assess soil bacterial and fungal beta diversity levels, and the results were visualized through principal coordinates analysis (PCoA) using the *vegan* package in R. Analysis of similarities was performed to quantitatively estimate community similarities among sample groups (Yan et al., 2020). Stacked bar charts illustrating the relative abundance of microorganisms in each group were drawn using Python ver. 2.7. The relative abundances of bacteria and fungi in soil samples from each land use type were compared using one-way analysis of variance, followed by Tukey’s multiple comparison test to detect significant differences. Spearman’s correlation analysis was conducted using the *psych* package in R, and a heatmap of the results was plotted using Python ver. 3.7. Variance partitioning analysis was performed and visualized using the *vegan* package in R. Soil environmental factors were categorized as basic physicochemical properties (pH, soil moisture, and electrical conductivity), soil nutrients (SOC, TN, AP, and AK content and AN decomposition) and microorganism nutrients (MBC and MBN).

### 2.6 Soil microbiological network analysis

A soil microbial community co-occurrence network was constructed based on ASV levels using the Spearman correlation method (r ≥ 0.7, false discovery rate-adjusted *p* < 0.05), implemented in the *igraph* R package (Zhou et al., 2011; Yuan et al., 2021). To minimize potential spurious correlations, we selected only ASVs occurring in ≥30% of all samples and accounting for >0.01% of the total for the correlation calculation. The Gephi ver. 0.10.1 software was used for network visualization. To interpret the effects of land use types on the complexity of ecological networks, we extracted network topological characteristics, including node number, edge number, average degree, average weighted degree, network diameter, network density, modularity, average clustering coefficient, and average path length, for each soil sample using the “subgraph” function in the *igraph* R package (Jiao et al., 2022).

## 3 Result

### 3.1 Effects of land use type on soil microbial community diversity

**Figure 1:**
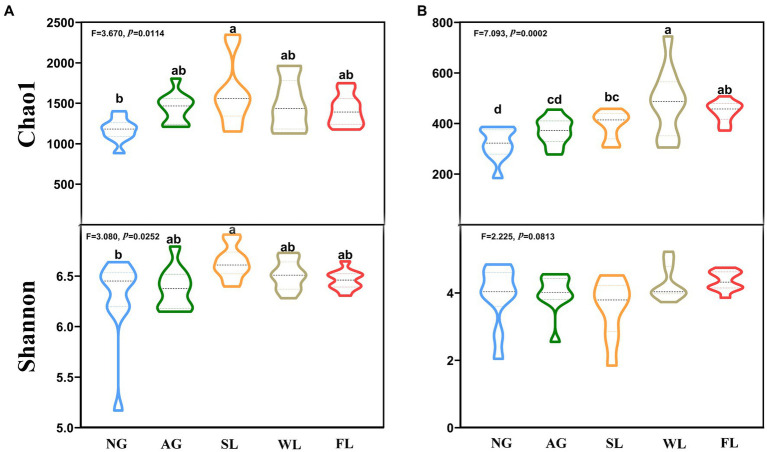
Indicators of α-diversity among soil **(A)** bacteria and **(B)** fungi in different land use types. Different letters indicate significant differences (analysis of variance followed by Tukey’s test; *p* > 0.05). AG, artificial grassland; MF, maize farmland; NG, natural grassland; SL, shrubland; WL, woodland.

We calculated changes in bacterial and fungal α-diversity for different land use types (Figures 1A,B). The results showed that bacterial richness (Chao1 index) and diversity (Shannon index) were highest in shrublands and lowest in natural grasslands. Woodlands showed the highest fungal abundance, and there were no significant differences in fungal diversity among groups.

**Figure 2:**
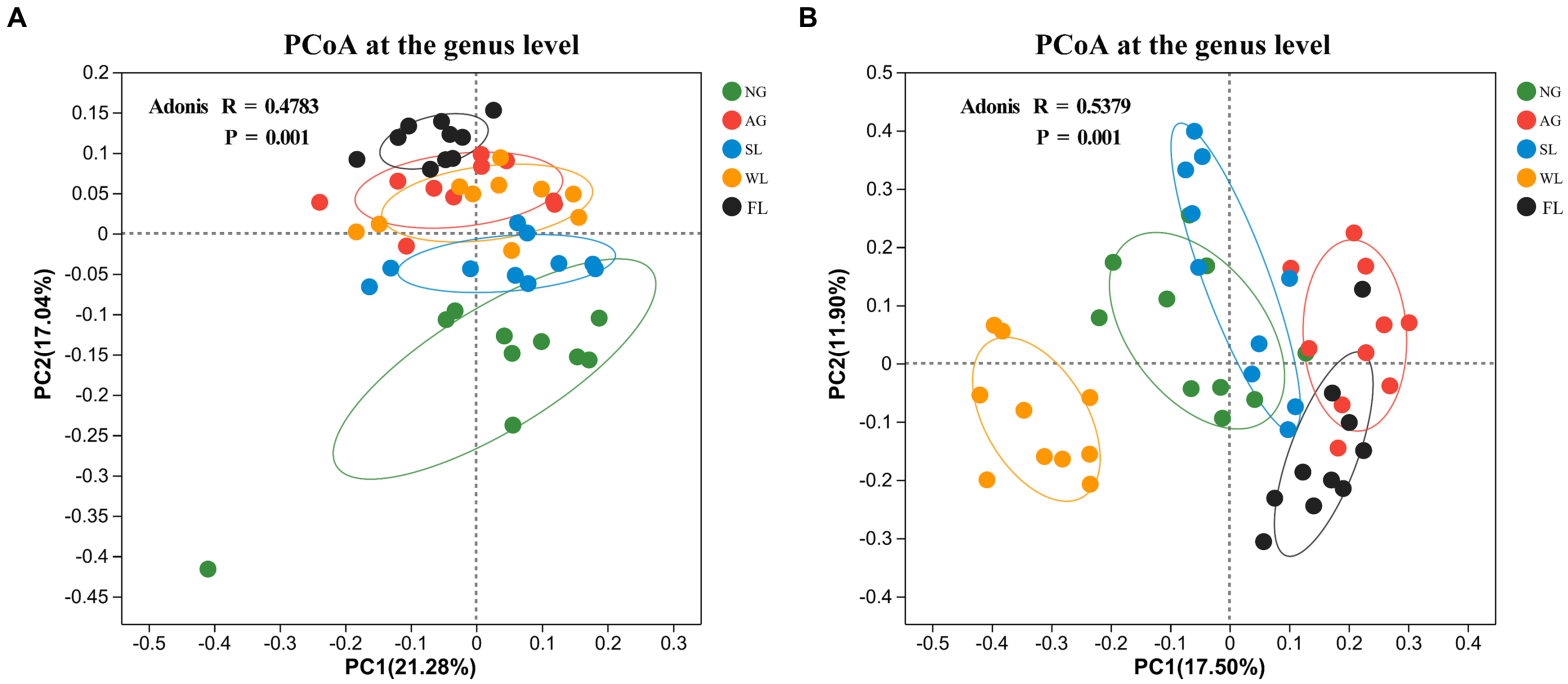
Principal coordinates analysis (PCoA) results for soil **(A)** bacterial and **(B)** fungal communities in different land use types. AG, artificial grassland; MF, maize farmland; NG, natural grassland; SL, shrubland; WL, woodland.

PCoA analyses of β-diversity based on the Bray–Curtis difference matrix showed clear separation of bacterial (*R* = 0.4783, *p* = 0.001) and fungal (*R* = 0.5379, *p* = 0.001) community structures across land use types (Figures 2A,B).

### 3.2 Effects of land use type on soil microbial community composition

**Figure 3:**
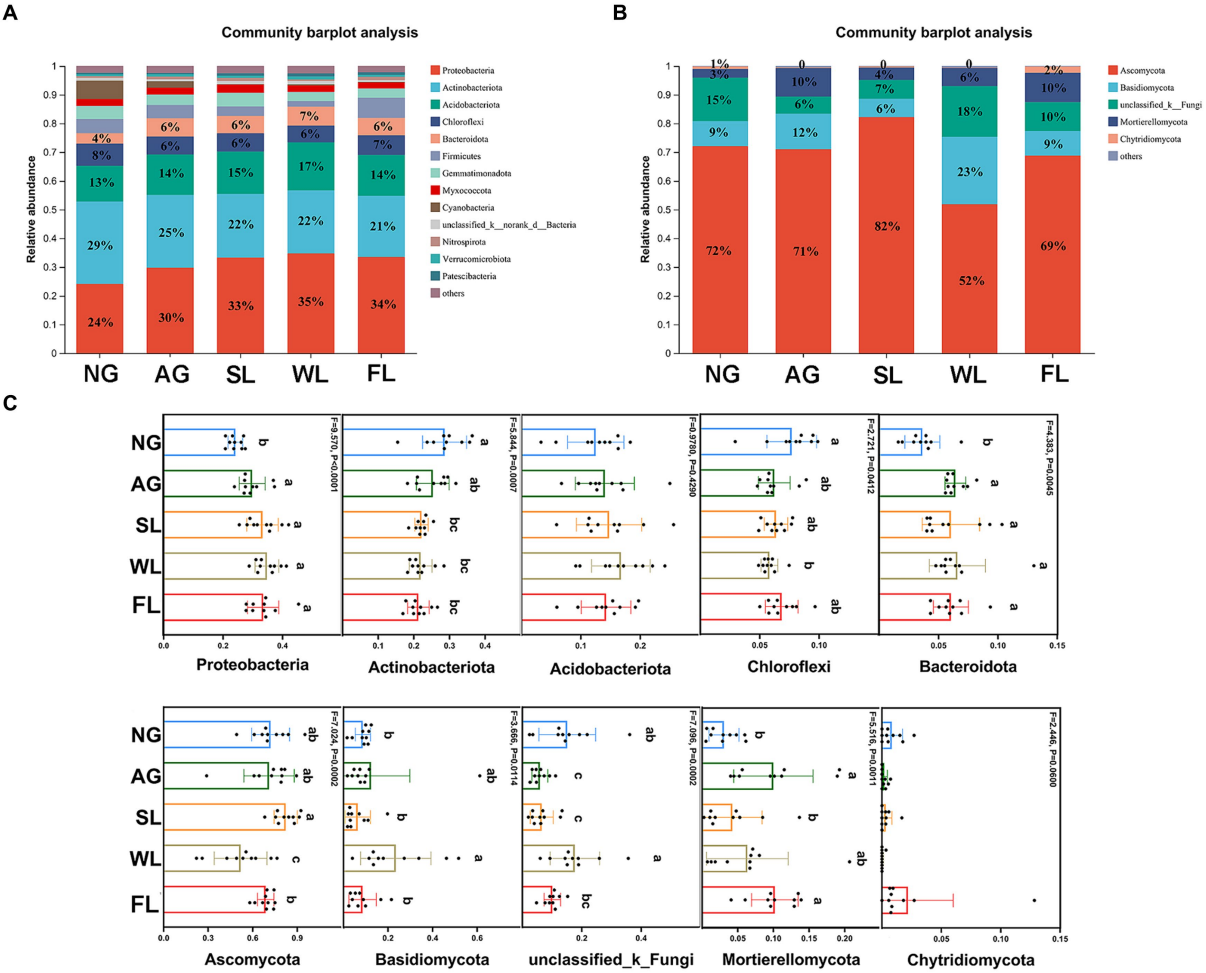
Relative abundance levels of the dominant **(A)** bacterial and **(B)** fungal phyla in soils sampled from different land use types. Relative abundance levels of the five most dominant **(C)** bacterial and **(D)** fungal taxa. Different letters indicate significant differences among land use types (analysis of variance followed by Tukey’s test; *p* > 0.05). AG, artificial grassland; MF, maize farmland; NG, natural grassland; SL, shrubland; WL, woodland.

Figure 3 shows bacterial and fungal community compositions at the phylum level under different land use types. Actinobacteriota had the highest relative bacterial abundance in natural grasslands, at 29%. In the other four land use types, Proteobacteria had the highest relative bacterial abundance, at 30–35%. Ascomycota had highest relative fungal abundance across all five land use types. The relative abundances of Proteobacteria and Bacteroidota were significantly lower in natural grasslands than in all other land use types. Actinobacteria had significantly higher relative abundance in natural grasslands than in shrublands, woodlands, and farmlands, and Chloroflexi had significantly higher relative abundance in natural grasslands than in woodlands. Among fungi, Ascomycota had significantly lower relative abundance in woodlands than in all other land use types, and the relative abundance of Basidomycota was significantly higher in woodlands than in natural grasslands, shrublands, and farmlands.

### 3.3 Effects of land use type on microbial community networks

**Figure 4:**
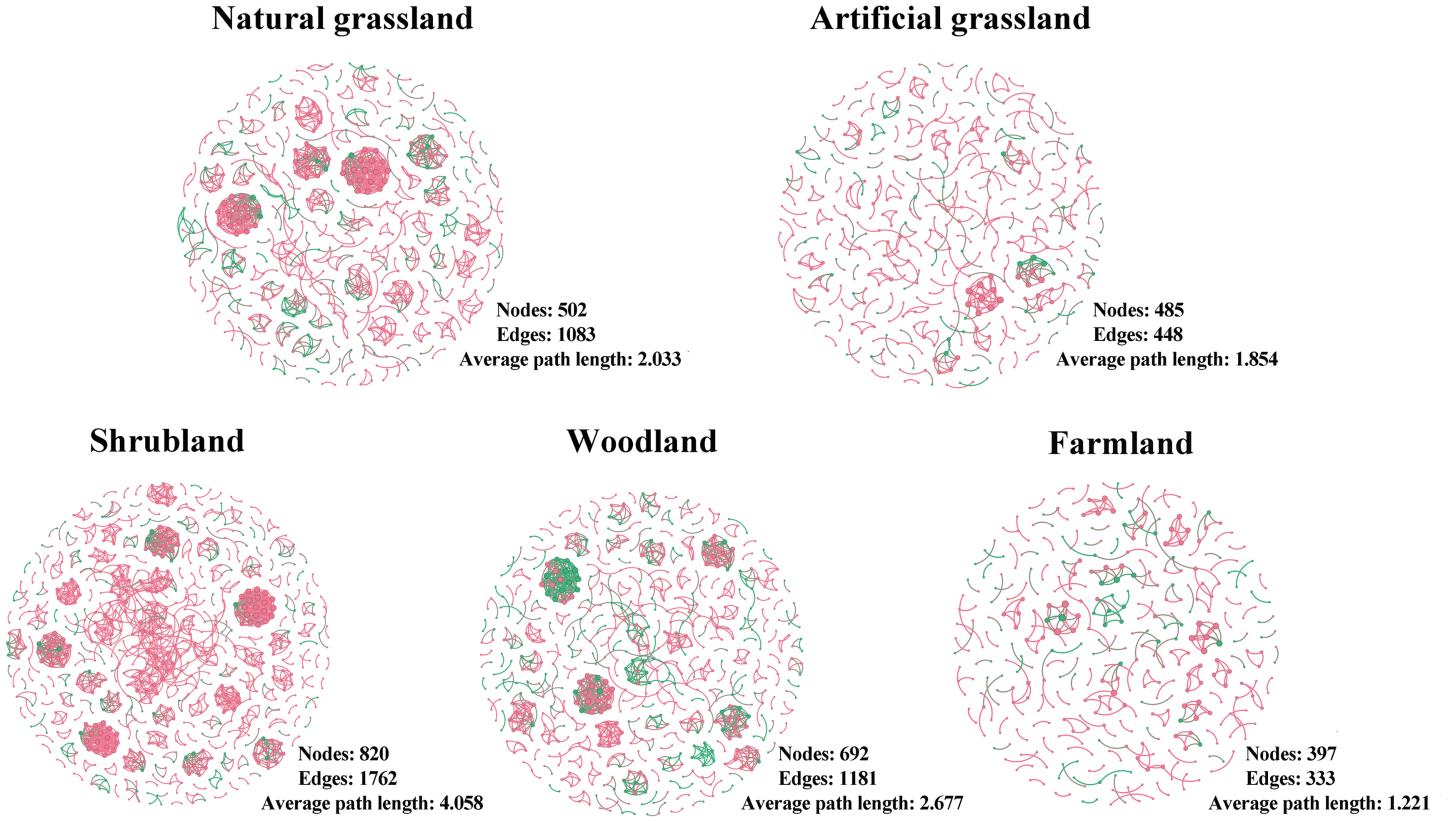
Soil microbial networks in the land use types examined in this study. Each network represents a random matrix theory co-occurrence model, where nodes represent amplicon sequence variants, red indicates bacteria, and green indicates fungi. Edges between nodes represent significant correlations.

We constructed microbial community networks for each of the five land use types and found that their structures varied greatly (Figure 4; Supplementary Table S2). Artificial grasslands and farmlands had simpler network structures, with fewer nodes and edges, and lower average degree, average weighted degree, mesh diameter, and average path length; by contrast, these metrics were higher and the network structures were more complex in natural grasslands, shrublands, and woodlands. There were no clear trends among land use types for indicators such as network density, degree of modularity, and average degree of clustering.

### 3.4 Correlation of soil microbial communities with environmental factors

**Figure 5:**
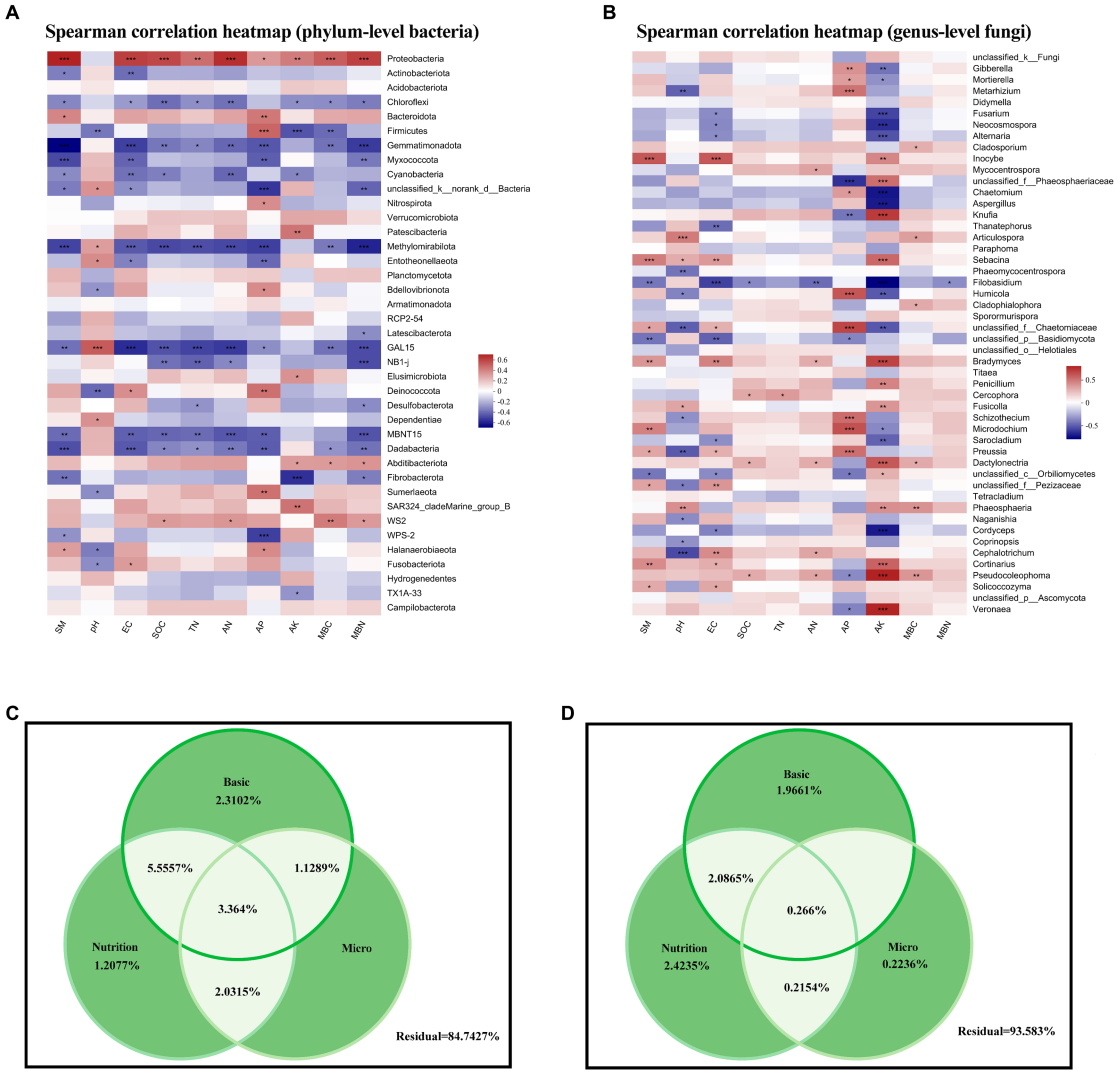
**(A,B)** Heatmaps of Spearman correlations between soil properties and **(A)** phylum-level bacterial abundance and **(B)** genus-level fungal abundance. **(C,D)** Variance partitioning analysis of the degree of individual and joint explanation of **(C)** bacterial and **(D)** fungal abundance according to basic physicochemical properties, soil nutrients, and microbial nutrients. SM, soil moisture; EC, electrical conductivity; SOC, soil organic carbon; TN, total nitrogen; AN, available nitrogen; AP, available phosphorus; AK, available potassium; MBC, microbial biomass carbon; MBN, microbial biomass nitrogen.

At the phylum level, Proteobacteria and Chloroflexi, which represented a large proportion of bacteria in our soil samples, were significantly correlated with various environmental factors, showing positive and negative correlations with the majority of environmental factors, respectively (Figure 5A). Gemmatimonadota, Methylomirabilota, Dadabacteria, and candidate phyla GAL15 and MBNT15 were also strongly (mainly negatively) correlated with environmental factors.

However, at the fungal genus level, AP and AK were positively or negatively correlated with most taxa (Figure 5B). We attempted to explain these differences in bacterial and fungal community structure according to environmental factors by dividing these into three categories: basic physicochemical properties (pH, soil moisture, and electrical conductivity), soil nutrients (SOC, TN, AP and AK content, and AN decomposition), and microorganism nutrients (MBC and MBN), but the results were not conclusive, with residuals of 84.73 and 93.58% for bacterial and fungal communities, respectively.

## 4 Discussion

We investigated differences in microbial community structure in the agro-pastoral ecotone of northern China, across a larger variety of land use types than examined in previous studies, including woodlands. The results showed significant differences in microorganism composition and diversity among land use types. The network structures of natural grasslands, shrublands, and woodlands were more complex, whereas those of artificial grasslands and croplands were simpler. Despite a degree of correlation between soil environmental factors and microorganism abundance in some taxa, our data were unable to explain differences among microbial communities, suggesting that anthropogenic activities may have had a greater influence than soil environmental factors on soil microbes.

### 4.1 Effects of land use type on soil microbial community diversity and composition

We detected significant differences in bacterial diversity and abundance among the five land use types, which may have been caused by differences in the dominant plants and degree of anthropogenic interference among land use types (Szoboszlay et al., 2017; Nkuekam et al., 2018). Bacterial richness and diversity index values were highest in shrublands, perhaps because shrubland vegetation has higher biomass, water status, and soil permeability, as well as denser root systems than the other land use types examined in this study (Michelsen et al., 1996; Guanghua et al., 2006). In addition, shrubland is also the least anthropogenic land use among the five land use types (no ploughing, watering, grazing, recreation, etc.), which may be one of the reasons for its higher diversity (Tomazelli et al., 2023). Fungal richness was also highest in woodlands, probably because tall trees have strong xylem root systems that can act as hosts for fungi and can provide them with available nutrients (Urbanova et al., 2015; de Vries et al., 2018). The α-diversity indices of both bacteria and fungi were lower in natural grasslands, presumably due to poorer soil conditions and lower moisture content (Supplementary Table S3), and because herbaceous root systems are less able to retain water (Torsvik and Ovreås, 2002; Deng, 2012). The PCoA results showed that soil bacterial and fungal β-diversity values were highly variable across land use types (Figures 2A,B; *p* = 0.001). We hypothesise that this is the result of large differences in anthropogenic activities under different land-use types, and that actions such as grazing (Wang and Tang, 2019), fertilizing (Zhong and Cai, 2004), and tilling (Fan et al., 2010) are all important measures to modify heterogeneity between microbiomes.

The phylum Actinobacteria had the highest bacterial relative abundance in natural grasslands, whereas Proteobacteria had the highest bacterial relative abundance in all other land use types. This result may be explained by the co-trophic hypothesis (Fierer et al., 2012). Actinomycetes acquire water and limited nutrients through hypha modification, which allows them to adapt to harsh conditions such as drought (Xu et al., 2021). Among the land use types examined in this study, natural grassland had the lowest soil water content and therefore the highest relative abundance of actinomycetes due to the shallowest root system and lowest height of the vegetation, and therefore poor soil water fixation and high evapotranspiration. Many members of the Proteobacteria are N-fixing; these bacteria can increase nutrient use efficiency by dissolving phosphates, fixing N, and degrading residues (Yang et al., 2023). Compared to natural grasslands, the other land use types exhibited higher N demands (Cornell et al., 2023), resulting in a significant increase in the relative abundance of Proteobacteria. In summary, differences among land use types within the agro-pastoral ecotone of northern China led to differences in microbial community composition and diversity.

### 4.2 Effects of land use type on soil microbial networks

We detected fewer nodes and edges and a lower average degree of network structure in artificial grasslands and farmlands, indicative of a simpler network structure (Lan et al., 2022). However, the mean path length and network diameter were also smaller in these land use types, suggesting that microorganisms in these networks were more closely connected to each other (Liu L. X. et al., 2023). In contrast to our results, a recent Brazilian study found more nodes and edges in the network structures of more intensively managed rangelands than in natural grasslands (Tomazelli et al., 2023). This discrepancy may be largely attributed to differences in climatic conditions between Brazil and the Inner Mongolian Plateau (Goss-Souza et al., 2017). Microbial network structural complexity is not only reflected in the numbers of nodes and edges but also closely related to the network diameter and average path length (Lan et al., 2022). However, we also found smaller network diameters and average path lengths in both artificial grasslands and farmlands, suggesting that microorganisms are more tightly connected and that materials and energy are less lost in the transfer process, making their efficient transfer possible (Liu L.X. et al., 2023). Consistent with the results of previous studies (Goss-Souza et al., 2017; Pedrinho et al., 2019; Costa et al., 2022; Cornell et al., 2023), we found that intensive land use can increase network structural complexity through reducing the network diameter and average path length, possibly because the land causes the death of many microorganisms during and intense use, and the remaining microorganisms need to co-operate better in order to adapt to the new environment (Cornell et al., 2023). However, the overall complexity did not change significantly due to decreased node and edge numbers.

In shrublands and woodlands, which are characterized by less anthropogenic disturbance, we observed the opposite trend, with more nodes and edges and smaller network diameters and average path lengths. Recent studies have also shown increases in the numbers of microbial network nodes and edges following land use shifts from grassland to woodland (Yang et al., 2022). This phenomenon coincides with the highest microbial diversity and richness in scrub and woodland observed in this study, with the highest number of nodes and edges due to more microbes involved in the network structure.

### 4.3 Soil properties are not important contributors to microbial community structural differences

Numerous studies have shown that soil-based physicochemical factors such as soil pH, moisture, and electrical conductivity have important effects on microbial communities (Wu et al., 2020; Chen et al., 2021; Bai et al., 2023). Soil microbial communities may also be affected by nutrients such as C, N, P, and K (Moro et al., 2014; Huang et al., 2016; Batista and Dixon, 2019; Ali et al., 2022), which regulate soil physicochemical properties and alter the inter-root microecological environment by influencing plant root secretions. We predicted that MBC and MBN would influence microbial structures. However, our results showed that although these metrics showed some correlation with the relative bacterial and fungal abundance (Figures 5A,B), the soil base physicochemical traits did not explain structural differences in bacterial and fungal communities (Figures 5C,D), perhaps because anthropogenic disturbance levels varied greatly between the different land use types examined in this study. Examples include occasional grazing on natural grasslands, compared to fertilizer and water application on artificial grasslands and farmlands, and recreation by villagers on woodlands.

Tillage can also impact soil fungal diversity (Liu W. S. et al., 2023). Artificial grasslands and farmlands with different tillage practices and higher tillage intensity levels due to increasing demand for higher agricultural yields may be important contributors to microbial community structural changes (Zhang et al., 2023). Thus, water and fertilizer management in artificial grasslands and farmlands may be an important reason for microbial community structural differences compared to natural grasslands, shrublands, and woodlands. Grazing affects the structure of subsurface microbial communities through animal feeding, trampling, and the return of feces and urine to soil (Ingram et al., 2008; Aldezabal et al., 2015; Zhao et al., 2017); therefore, grazing behaviors may explain microbial community differences between natural grasslands and other land use types. To summarize, our results showed that soil properties had a limited influence on microbial community structure in the agro-pastoral ecotone of northern China. Thus, we speculate that differences in anthropogenic activity levels among the land uses types examined in this study had the most important influence on microbial community structure in the study area, in contradiction to our second hypothesis.

## 5 Conclusion

Our results provide new insights into soil microbial community structural variation under different land use types in the agro-pastoral ecotone of northern China. We detected differences in soil microbial diversity, composition, and network structure under different land use types in the study region. Soil properties were correlated with the relative abundances of certain microorganisms, but explained only a small part of the variation in bacterial and fungal community structure. This suggests that variations in anthropogenic activity intensity among land use types may have a greater influence on microbial community structure.

## Data availability statement

The original contributions presented in the study are publicly available. This data can be found at: NCBI (https://www.ncbi.nlm.nih.gov/), accession number: PRJNA1068828.

## Author contributions

ZS: Conceptualization, Data curation, Formal analysis, Investigation, Supervision, Writing – original draft, Writing – review & editing. CS: Data curation, Formal analysis, Writing – original draft. TZ: Investigation, Supervision, Writing – review & editing. JL: Data curation, Formal analysis, Writing – original draft. XW: Data curation, Formal analysis, Writing – original draft. JF: Data curation, Formal analysis, Writing – original draft. SL: Investigation, Supervision, Writing – review & editing. ST: Conceptualization, Funding acquisition, Investigation, Resources, Supervision, Writing – original draft, Writing – review & editing. KJ: Conceptualization, Funding acquisition, Investigation, Resources, Supervision, Writing – review & editing.
