# Supplementary material for: Soil microbial community variation among different land use types in the agro-pastoral ecotone of northern China is likely to be caused by anthropogenic activities
Source: Front Microbiol. 2024 May 22;15:1390286. doi: 10.3389/fmicb.2024.1390286 (PMC11150776; doi:10.3389/fmicb.2024.1390286)
Supplement: Supplementary file 1 [file Data_Sheet_1.pdf]

Supplementary Information for

Soil microbial community variation among different land use types in the agro-pastoral ecotone of northern China is likely to be caused by anthropogenic activities

Zhaokai Sun[a], Chongzhi Sun[a], Tongrui Zhang[a], Jia Liu[c], Xinning Wang, Jing Feng[c], Shiming Tang[a*], Ke Jin[a,b*]

[a] Key Laboratory for Model Innovation in Forage Production Efficiency, Ministry of Agriculture and Rural Affuirs, Institute of Grassland Research, Chinese Academy of Agricultural Sciences, Hohhot 010010, China

[b] Department of International Cooperation, Chinese Academy of Agricultural Sciences, Beijing 100081, China

[c] School of Grass Academy, Qingdao Agriculture University, Qingdao 266109, China

* Corresponding author: Shiming Tang, E-mail: tangsm001@126.com; Ke Jin, E-mail: jinke@caas.cn

Table S1 Geographical features and vegetation of different study sites.

Land-use type	Abbreviations	Coordinates	Vegetation type	Dominant species	Mean tree/crop height (m)	Water Fertilizer Addition	Tillage
Natural grassland	NG	N40°35′25″, E111°46′59″	*Stipa capillata L, Leymus chinensis, Agropyron cristatum*, etc	*Stipa capillata L*	0.3	No	No
Artificial grassland	AG	N40°35′15″, E111°46′56″	*Leymus chinensis*	*Leymus chinensis*	0.5	Yes	Yes
Shrubland	SL	N40°35′22″, E111°46′47″	*Caragana korshinskii Kom*, a few weeds	*Caragana korshinskii Kom*	1.5	No	No
Woodland	WL	N40°35′19″, E111°46′46″	*Populus L., Setaria viridis (L.) P. Beauv*, etc	*Populus L.*	over 3	No	No
Farmland	FL	N40°35′10″, E111°46′46″	*Zea mays L.*	*Zea mays L.*	2.2	Yes	Yes

Table S2 Key topological properties of bacterial-fungal co-occurrence networks under different land uses.

Topological properties	NG	AG	SL	WL	FL
nodes	602	485	820	692	397
(Bacteria and Fungi nodes /%)	75.1, 24.9	79.6, 20.4	83.4, 26.6	68.5, 31.5	77.1, 22.9
edges	1083	448	1762	1181	333
(Positive and Negative edges /%)	99.2, 0.8	97.3, 2.7	97.3, 2.7	97.6, 2.4	98.2, 1.8
average degree	3.598	1.847	4.298	3.413	1.678
average weighted degree	3.481	1.809	4.156	3.305	1.649
network diameter	9	4	13	11	3
network density	0.006	0.004	0.005	0.005	0.004
modularity	0.954	0.982	0.951	0.962	0.984
average clustering coefficient	0.773	0.708	0.755	0.712	0.805
average path length	2.033	1.354	4.058	2.677	1.221

Table S3 Mean values of soil parameters under five different land uses (n=10).

Soil parameters	NG	AG	SL	WL	FL
SM (%)	7.64±0.52b	8.63±0.85b	8.44±0.61b	12.34±0.91a	12.88±0.48a
pH	8.30±0.03b	8.44±0.03a	8.27±0.02b	8.42±0.02a	8.00±0.03c
EC (us/cm)	101.4±4.14c	95.24±1.78c	113.80±5.92bc	131.52±6.57b	159.93±7.39a
SOC (g/kg)	6.07±0.48a	6.05±0.35a	8.10±0.99a	7.89±0.93a	6.44±0.31a
TN (g/kg)	0.60±0.05a	0.55±0.03a	0.77±0.10a	0.66±0.08a	0.64±0.04a
AN (mg/kg)	43.75±4.10a	43.47±2.44a	61.50±6.69a	56.04±6.74a	52.92±1.83a
AP (mg/kg)	4.20±0.45b	8.6±2.67b	4.75±0.33b	4.19±0.28b	16.25±2.13a
AK (mg/kg)	102.93±15.34b	70.26±4.01b	151.74±13.94b	433.72±47.57a	77.32±3.22b
MBC (mg/kg)	84.60±10.15a	91.52±4.35a	118.54±20.42a	139.39±27.75a	87.50±10.38a
MBN (mg/kg)	12.42±1.71a	14.46±1.66a	17.94±3.29a	20.14±4.09a	14.86±1.30a

Abbreviations: SM, soil moisture content; pH, pH value; EC, electrical conductivity; SOC, soil organic carbon; TN, total nitrogen; AN, alkaline nitrogen decomposition; AP, available phosphorus; AK, available potassium; MBC, microbial biomass carbon; MBN, microbial biomass nitrogen. Data represent means and S.E. Different lowercase letters across rows indicate statistically significant differences at $P < 0.05$.

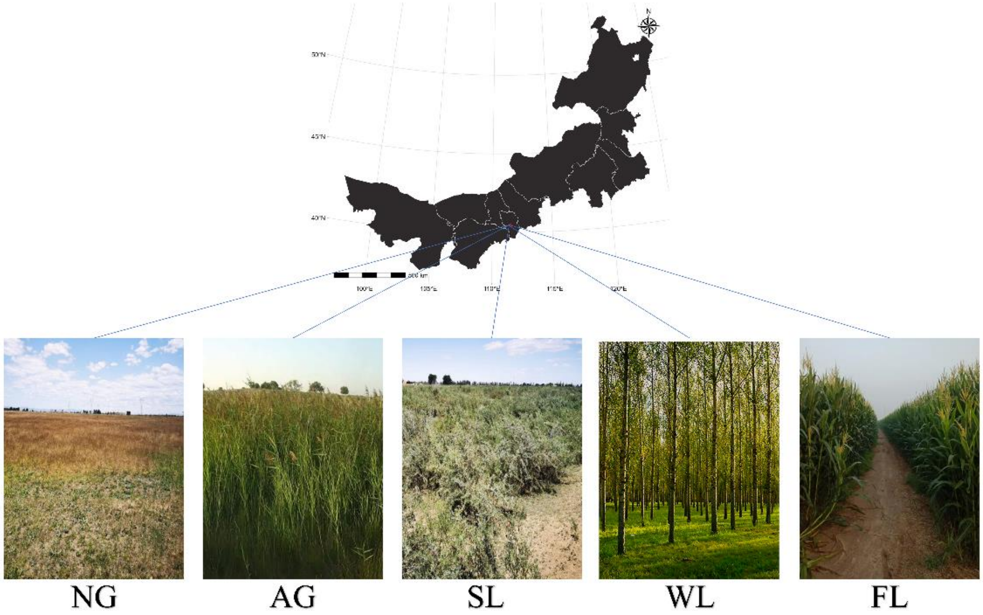

Fig. S1. The study sites and representative vegetations: Natural grassland (NG); Artificial grassland (AG); Shrubland (SL); Woodland (WL); farmland (FL).
